# Cholangiocarcinoma: Is Preoperative Histologic Confirmation Mandatory Before Major Hepatobiliary Surgery?

**DOI:** 10.7759/cureus.103788

**Published:** 2026-02-17

**Authors:** Sergio Isidro Gamboa-Hoil

**Affiliations:** 1 Surgical Oncology, Mexican Social Security Institute, Merida, MEX

**Keywords:** bile duct neoplasms, biliary tract surgical procedures, biopsy, cholangiocarcinoma, diagnosis, hepatectomy

## Abstract

Cholangiocarcinoma is an aggressive malignancy of the biliary epithelium frequently diagnosed at advanced stages. Establishing a definitive preoperative histologic diagnosis remains challenging, particularly in perihilar and infiltrative tumors, as currently available diagnostic techniques may not reliably exclude malignancy. Although positive cytologic and biopsy results strongly support the presence of cancer, negative findings do not consistently rule it out. This diagnostic uncertainty raises an important clinical question regarding whether histologic confirmation is mandatory before major hepatobiliary resection. A narrative literature review was conducted using the PubMed/MEDLINE, Scopus, and Web of Science databases, including studies published in English between January 1990 and January 2026. Relevant articles evaluating diagnostic modalities, cytologic accuracy, surgical management, perioperative outcomes, and survival in patients with suspected or confirmed cholangiocarcinoma were reviewed. Available evidence demonstrates substantial limitations in preoperative tissue diagnosis. Imaging modalities and tumor markers provide supportive but non-definitive diagnostic information, while biliary cytology and biopsy remain suboptimal for definitively excluding malignancy. Surgical series consistently report that a proportion of patients undergoing resection for presumed malignancy ultimately have benign disease on final pathology; nevertheless, outcomes following timely surgical exploration and resection in appropriately selected patients appear superior to those of patients managed without surgery. Resection remains the only established potentially curative treatment. Preoperative histologic confirmation may not be mandatory in carefully selected patients with high clinical and radiologic suspicion of cholangiocarcinoma and technically resectable disease. Timely surgical intervention within a multidisciplinary framework, particularly in experienced hepatobiliary centers, may help avoid loss of curative opportunity.

## Introduction and background

Cholangiocarcinoma is an aggressive malignant neoplasm arising from the biliary epithelium that can involve any segment of the biliary tree, from the intrahepatic bile ducts to the distal common bile duct at the level of the duodenal ampulla [1-3]. It accounts for approximately 3% of all gastrointestinal malignancies, represents the second most common primary hepatic tumor, and comprises about 10%-15% of all hepatobiliary cancers [2,3].

Anatomic classification and tumor distribution

**Figure 1:**
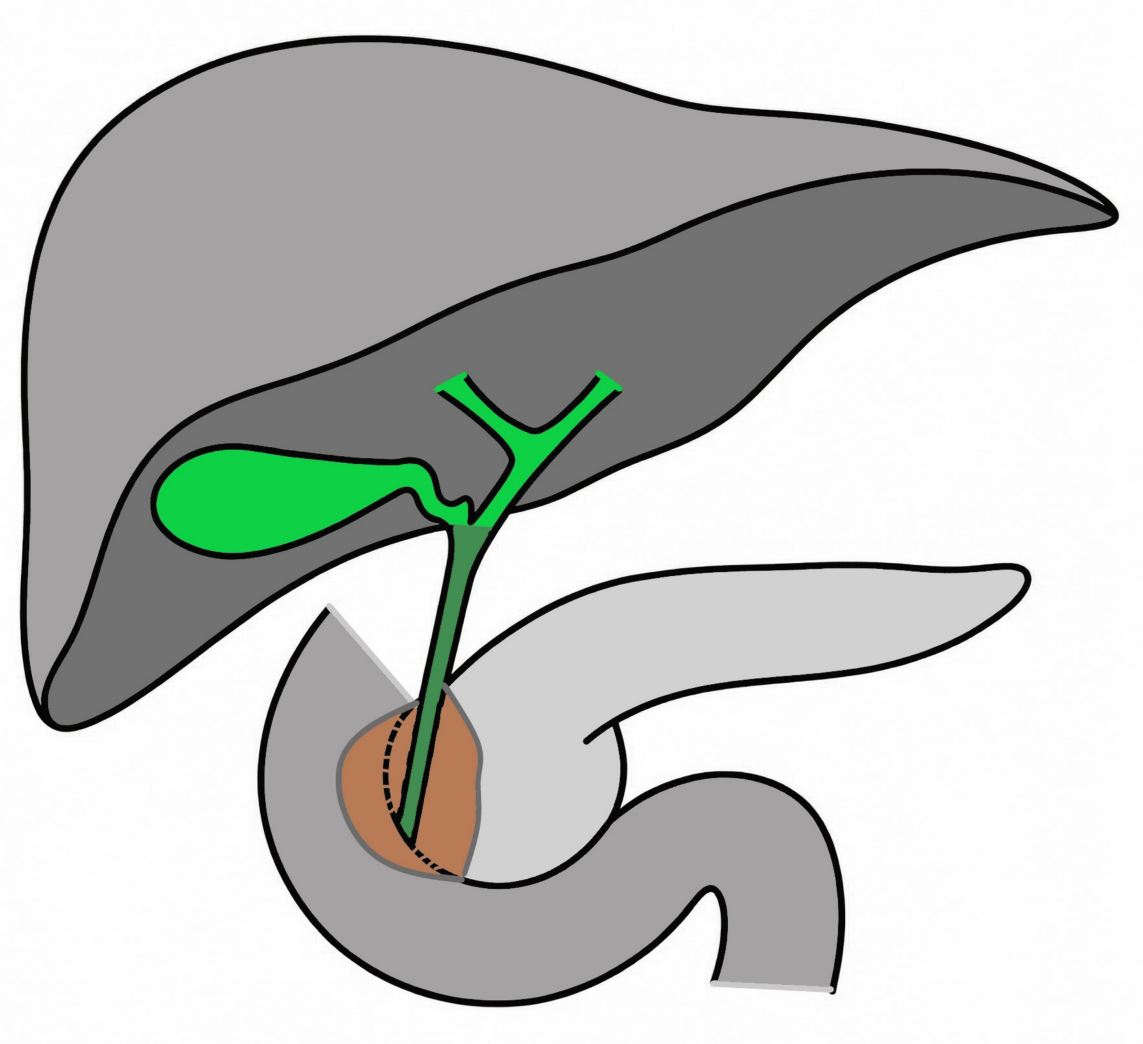
Schematic representation of the hepatobiliary anatomy The intrahepatic bile ducts are not individually depicted as they course within the liver parenchyma. The hilar bile duct is shown in green, while the distal bile duct is highlighted in a darker green to emphasize its intramural course. The brown-shaded area represents a sectional anatomical view of the intrapancreatic and duodenal wall tissues through which the distal bile duct traverses and does not depict a tumor or pathologic lesion. Image credit: Original illustration created by the authors.

Among patients who underwent surgical exploration, intrahepatic cholangiocarcinoma accounted for 55.3% of cases, followed by perihilar cholangiocarcinoma (32.9%) and distal cholangiocarcinoma (11.8%) (Figure 1) [4].

Clinical characteristics and presentation

The disease occurs more frequently in men, accounting for approximately 60-66% of cases, and is typically diagnosed between the late fifth and seventh decades of life in contemporary series [5-8].

The clinical presentation is predominantly characterized by obstructive jaundice, reported in approximately 75-90% of patients in contemporary series. Other common symptoms include weight loss (approximately 40-45%), abdominal pain (40-50%), anorexia (around 55%), and fever (approximately 10-15%) [4,6,9].

Tumor markers

Carbohydrate antigen 19-9 (CA 19-9) is the most widely used serum biomarker in cholangiocarcinoma. At a commonly applied cutoff value of 37 U/mL, reported diagnostic performance includes a sensitivity of 77.1%, specificity of 84.8%, positive predictive value of 65.9%, and negative predictive value of 90.7%. However, CA 19-9 levels may be elevated in benign biliary obstruction and inflammatory conditions, thereby limiting specificity. Additionally, the marker is undetectable in approximately 5-10% of individuals who are Lewis antigen-negative. Therefore, although elevated CA 19-9 levels may support clinical suspicion in the appropriate setting, the biomarker lacks sufficient sensitivity and specificity to serve as a definitive standalone diagnostic tool [10,11].

Diagnostic imaging and endoscopic modalities

Cross-sectional imaging is fundamental in the evaluation of suspected cholangiocarcinoma. Ultrasonography is typically the first-line modality, whereas CT is primarily used for staging and vascular assessment. MRI remains the most informative non-invasive technique for detailed biliary mapping [12].

Endoscopic and percutaneous approaches provide both diagnostic and therapeutic capabilities. Endoscopic retrograde cholangiopancreatography (ERCP) and percutaneous transhepatic cholangiography (PTC) allow tissue sampling and drainage but are invasive. Advanced modalities, such as direct single-operator cholangioscopy (DSOC) and endoscopic ultrasound-guided fine-needle aspiration (EUS-FNA), enhance diagnostic accuracy in selected scenarios [12,13].

The reported diagnostic performance of these modalities is summarized in Table 1.

**Table 1:** Diagnostic modalities in suspected cholangiocarcinoma Abbreviations: US, ultrasonography; CT, computed tomography; MRI, magnetic resonance imaging; ERCP, endoscopic retrograde cholangiopancreatography; PTC, percutaneous transhepatic cholangiography; DSOC, direct single-operator cholangioscopy; EUS-FNA, endoscopic ultrasound–guided fine-needle aspiration.

Modality	Sensitivity (%)	Specificity (%)
US	87 -96	Not consistently reported
CT	61	88
MRI	90-97	60-81
ERCP/PTC	46-73	Approaching 100 (with biopsy)
DSOC	85-86	Approaching 100
EUS-FNA	43-89	79-100

Cytologic evaluation of biliary lesions

Biliary strictures are frequently not amenable to conventional core biopsy; therefore, cytologic techniques, such as brush cytology and bile aspirate analysis, are commonly employed. Reported sensitivity for ERCP-guided brush cytology ranges widely (approximately 26-57%), whereas specificity consistently approaches 97-100%. In large historical series, sensitivity has been reported around 60%, with specificity exceeding 98%, yielding a high positive predictive value but a limited negative predictive value. Contemporary analyses continue to report modest sensitivity despite consistently high specificity [13,14].

These characteristics underscore the central diagnostic limitation: while a positive cytologic result strongly supports malignancy, a negative result does not reliably exclude it. In addition, bile aspirate cytology is often suboptimal because exfoliated tumor cells may rapidly degenerate in bile, reducing cellular preservation and diagnostic yield. Furthermore, although uncommon, false-positive cytologic interpretations and potential tumor cell displacement during sampling have been described [13,14].

Clinical rationale for upfront resection without histologic confirmation

Surgical resection remains the only established potentially curative treatment for cholangiocarcinoma. However, preoperative tissue confirmation is often challenging due to the limited sensitivity of brush cytology, forceps biopsy, and fine-needle aspiration, largely attributable to the desmoplastic and submucosal growth pattern characteristic of these tumors. Consequently, negative biopsy results do not reliably exclude malignancy in patients with a high degree of clinical and radiologic suspicion [13-15].

In high pretest probability scenarios, particularly in patients presenting with dominant hilar strictures, progressive obstructive jaundice, and characteristic cross-sectional imaging findings, the overall likelihood of malignancy is substantial. Although brush cytology demonstrates high specificity (approximately 98%) and positive predictive value (95-98%) when positive, its sensitivity remains limited (around 55-60%), resulting in a relatively low negative predictive value in this context. Consequently, histologic confirmation may remain inconclusive despite a strong clinical and radiologic suspicion of malignancy [5,13,14].

Repeated diagnostic attempts may delay definitive surgical management, and prolonged diagnostic pathways have been described in patients undergoing evaluation for suspected biliary malignancy. In locally aggressive tumors such as perihilar cholangiocarcinoma, interval disease progression during this period may adversely affect resectability and decrease the likelihood of achieving an R0 resection [1,4].

Tumor seeding following biopsy of cholangiocarcinoma has been reported, most notably in association with percutaneous transhepatic approaches. Although the absolute incidence appears low, cases of tumor tract implantation and peritoneal dissemination have been described after transperitoneal sampling techniques. Recent literature has revisited this issue and highlighted its potential oncologic implications, particularly in patients being considered for curative-intent resection. Reflecting these concerns, current guidelines indicate that tissue confirmation is not routinely required in patients with resectable disease and high clinical suspicion of cholangiocarcinoma [3,10,11,16,17].

Current international guidelines state that biopsy is usually not required in patients undergoing resection for suspected cholangiocarcinoma when clinical and radiologic findings are strongly suggestive of malignancy, and the tumor is technically resectable. Therefore, in carefully selected patients, upfront surgical exploration without mandatory histologic confirmation may represent a rational and oncologically sound strategy [3,11,15].

## Review

Search strategy and methods

A narrative literature review was conducted to evaluate the role of histologic confirmation in the surgical management of cholangiocarcinoma. A structured search of PubMed/MEDLINE, Scopus, and Web of Science was performed for articles published in English between January 1990 and January 2026. The selected time frame was intended to encompass the modern era of hepatobiliary surgical management, including the development of extended hepatic resections and contemporary diagnostic strategies. Search terms included combinations of “cholangiocarcinoma,” “biliary tract cancer,” “hilar cholangiocarcinoma,” “biliary stricture,” “histologic diagnosis,” “preoperative diagnosis,” “cytology,” “hepatectomy,” “pancreatoduodenectomy,” and “surgical outcomes.” Articles were selected based on clinical relevance to diagnostic decision-making and surgical management. Reference lists of key publications and major consensus statements were manually reviewed to identify additional seminal studies.

Inclusion and Exclusion Criteria

Studies were considered for inclusion if they involved patients with suspected or confirmed cholangiocarcinoma and addressed diagnostic modalities, histologic confirmation, or surgical management, including reporting of clinical, pathological, or survival outcomes. Case series, retrospective cohort studies, and relevant review articles were included when deemed informative to the clinical question. Case reports, non-human studies, non-English publications, and articles lacking meaningful clinical or surgical outcome data were excluded.

Risk-of-Bias Consideration

Given the narrative nature of this review and the predominance of retrospective studies in the available literature, a formal risk-of-bias assessment using standardized evaluation tools was not performed. Nevertheless, potential sources of bias, including selection bias, referral bias, and diagnostic verification bias, were considered during data interpretation. Particular attention was paid to heterogeneity in diagnostic criteria, surgical indications, and outcome reporting across studies when synthesizing conclusions.

Surgical assessment and resectability

Reported resectability rates reached approximately 83%, with curative-intent resections performed in up to 94% of patients undergoing surgical exploration. In that cohort, major hepatic resection was required in 87.4% of cases, reflecting the advanced stage at presentation and the technical demands of achieving oncologic clearance [4].

Across reported series, mean operative times have ranged from approximately 648 to 797 minutes. Intraoperative blood loss has been reported between 1,368 and 4,132 mL. Reported transfusion requirements vary across studies, with mean transfusion volumes of approximately 529 mL in selected series [18,19]. The median postoperative hospital stay was 25 days [7].

Hepatectomy

The distribution of right- and left-sided hepatectomy varies across reported series. Right-sided resections have accounted for approximately 33%-56% of procedures, whereas left-sided resections have represented 44%-66%, depending on tumor extent and institutional preference [7,8].

In more detailed multicenter analyses, extended right hepatectomy comprised 34% of cases, followed by standard left hepatectomy (32%), standard right hepatectomy (18%), and extended left hepatectomy (16%) [20].

Postoperative morbidity and mortality

Postoperative morbidity has ranged from 63% to 81% across reported series [20,21]. In a detailed, clinically relevant postoperative study, bile leakage occurred in 29% of patients, post-hepatectomy liver failure in 29%, and postoperative hemorrhage in 8.5% [21]. Reoperation has been reported in up to 12.3% of patients in selected series [19].

Thirty-day mortality has been reported at approximately 3.8% in selected series [19]. Reported 90-day mortality ranges from 10% to 14% in contemporary cohorts [6,21]. The most common causes of postoperative mortality include post-hepatectomy liver failure (6%), sepsis (3%), and hemorrhagic shock (1.5%) [21].

Histopathological findings and diagnostic accuracy

Final pathological analysis confirms cholangiocarcinoma in approximately 90% of resected cases in contemporary series. In another series, postoperative histopathological evaluation revealed no evidence of malignancy in approximately 7.2% of patients undergoing resection for suspected biliary malignancy. In an additional 2% of cases, a malignant neoplasm distinct from the preoperatively suspected perihilar cholangiocarcinoma was identified [6,22].

Among patients with a preoperative diagnosis of malignant biliary stricture, the false-positive rate reached 24.4% [22]. In another series of patients with suspected hilar malignancy, 13 cases were attributed to idiopathic benign strictures. Among these, 5 (38%) demonstrated chronic fibrosing inflammation, and 8 (62%) erosive inflammatory changes on histological examination. An additional 13 patients were found to have intervention-related strictures, most commonly following cholecystectomy (9 cases, 69%). Isolated cases were observed after prior radiotherapy, biliodigestive anastomosis, pancreaticoduodenectomy, and selective hepatic chemoembolization (1 case each, 8%) [15].

Histological Subtypes and Pathologic Stage

The median tumor size ranged from 3.6 to 4 cm across contemporary series [4,9]. Adenocarcinoma represents the predominant histological subtype of cholangiocarcinoma, accounting for approximately 95%-98% of cases, whereas adenosquamous carcinoma comprises a small minority, typically around 1%-2% [9,18].

Stage III disease was observed in 55.1% of patients, followed by stage II in 26.3% and stage IV in 15.7%. Lymph node metastasis was present in 51.5% of cases, lymphatic permeation in 44.9%, and perineural infiltration in 44.1% [4].

Surgical margin status and tumor spread

Negative surgical margins (R0) have been reported in approximately 52%-90% of cases across published series, whereas microscopically positive margins (R1) have been observed in up to 33% of patients [4,8].

In detailed surgical analyses, hepatic-side R1 margins occurred in 18.2% of patients and transverse R1 margins in 30.3% [23]. Macroscopic residual disease (R2) was identified in approximately 2.6% of cases [24].

The distance from the main tumor margin to the leading edge of tumor spread was greater in the hepatic direction (7.3 ± 3.5 mm) than in the duodenal direction (3.4 ± 1.6 mm); however, this difference was not statistically significant [23].

Overall and disease-free survival

Overall survival was 80.3% at 1 year, 37.6% at 3 years, and 22.9% at 5 years in a contemporary comparative series [8]. Reported overall survival following left-sided hepatectomy varies across contemporary series, with 3-year OS rates ranging from approximately 35% to 62% and 5-year OS from 20% to 30%. In patients undergoing right-sided hepatectomy, 3-year OS has been reported between 43% and 51%, and 5-year OS between 28% and 46% [7,8].

Three-year disease-free survival has been reported at approximately 60% following left-sided hepatectomy and 50% after right-sided hepatectomy in selected cohorts [7].

Recurrence

Among patients undergoing radical hepatectomy, 70.9% developed recurrence during follow-up. Recurrence occurred within 1 year in 26.0% of patients, between 1 and 3 years in 32.2%, between 3 and 5 years in 9.3%, and beyond 5 years in 3.4% of cases [25].

Prognostic Factors Influencing Survival

Lymph node metastasis (HR 2.372; 95% CI 1.208-4.657; p = 0.012), distant metastasis (HR 3.041; 95% CI 1.049-8.816; p = 0.041), and positive surgical margins (R1/R2) (HR 3.100; 95% CI 1.816-5.293; p < 0.001) were independently associated with reduced overall survival [4].

Outcomes Without Surgical Resection

Overall, 16.5% of patients were deemed unresectable at presentation. Among these patients, peritoneal carcinomatosis accounted for 51.0% of cases, locally advanced disease for 33.3%, and distant metastasis for 10.3% [4].

In early surgical series, 30-day mortality among unresectable patients who underwent exploratory laparotomy reached 50%, compared with 14.3% among those managed non-operatively [26].

In contemporary cohorts, median survival was 3.0 months for patients undergoing palliative-intent resection and 2.6 months for unresectable cases, without a significant difference between groups (p = 0.549) [4].

These findings suggest that, in patients with unresectable disease, palliative surgery does not confer a meaningful survival advantage over non-surgical management [4].

Role of adjuvant and neoadjuvant therapy

Radiotherapy is not routinely recommended after complete (R0) resection but may be considered, typically as chemoradiotherapy, in patients with R1/R2 margins or selected cases with nodal involvement [3,11,17].

Adjuvant chemotherapy is recommended following surgical resection, particularly in patients with high-risk pathological features such as positive margins or lymph node metastases. Neoadjuvant therapy may be considered in borderline resectable tumors within a multidisciplinary framework [3,11,17].

Limitations

This review has several limitations. First, as a narrative review, it does not follow a systematic review or meta-analysis methodology, which may introduce selection bias and limit reproducibility; however, this approach allowed the inclusion of both historical and contemporary studies relevant to surgical decision-making in cholangiocarcinoma. Second, the available evidence is predominantly derived from retrospective single-center series, which are subject to selection and referral biases, reflecting the rarity of the disease and the ethical constraints of randomized trials. Third, substantial heterogeneity exists among studies regarding diagnostic criteria, imaging techniques, surgical indications, and outcome definitions, limiting direct comparison and quantitative synthesis. Additionally, no pooled statistical analysis or meta-regression was performed, and survival outcomes are reported as described in the original publications. Finally, diagnostic performance estimates may be affected by verification bias, and the generalizability of older series to current practice may be limited by advances in diagnostic and perioperative management. Moreover, many reported outcomes originate from high-volume hepatobiliary centers, which may limit extrapolation to lower-volume institutions.

## Conclusions

Preoperative histologic confirmation of cholangiocarcinoma remains challenging, given the diagnostic uncertainty associated with currently available tissue sampling techniques. In carefully selected patients with high clinical and radiologic suspicion and technically resectable disease, the absence of definitive tissue diagnosis should not automatically preclude surgical exploration. Surgical resection continues to represent the only established potentially curative treatment and should be considered within a multidisciplinary framework, particularly in experienced hepatobiliary centers.

Although benign pathology may occasionally be identified on final histologic examination, current evidence suggests that outcomes associated with timely surgical management in appropriately selected patients may outweigh the risks of diagnostic delay. Further prospective studies are warranted to better define optimal decision-making algorithms in this complex clinical scenario.
